# Genome Sequence of *Salegentibacter mishustinae* KCTC 12263, Containing a Complete Subtype I-B CRISPR-Cas System

**DOI:** 10.1128/genomeA.00267-16

**Published:** 2016-04-14

**Authors:** Fei Zhang, Wenxin Lin, Rui Zhang, Qiang Zheng, Nianzhi Jiao

**Affiliations:** State Key Laboratory of Marine Environmental Science, Institute of Marine Microbes and Ecospheres, Xiamen University, Xiamen, People’s Republic of China

## Abstract

*Salegentibacter mishustinae* KCTC strain 12263 was isolated from the sea urchin *Strongylocentrotus intermedius* inhabiting the Sea of Japan. Here, we report the draft genome sequence of *Salegentibacter mishustinae* KCTC 12263. It comprises ~3.78 Mb in 38 contigs with a G+C content of 36.5%, and a total of 3,490 proteins-coding genes were obtained. One complete CRISPR-Cas gene cluster was identified in the genome, which shows the strategy against invasive genetic elements of the strain.

## GENOME ANNOUNCEMENT

Genus *Salegentibacter*, first named by McCammon and Bowman (1), belongs to the family *Flavobacteriaceae* of the phylum *Bacteroidetes*. Strains in the *Salegentibacter* genus share the characteristics of moderately halophilic, aerobic, Gram-negative stains that are nonmotile or motile by gliding, yellow-pigmented, and take MK-6 as the major isoprenoid quinine (1–4). Here, we report the draft genome of *Salegentibacter mishustinae* KCTC 12263, isolated from the sea urchin *Strongylocentrotus intermedius* inhabiting the Sea of Japan (5).

Strain *Salegentibacter mishustinae* was bought from the Korean Collection for Type Culture (KCTC). Whole-genome shotgun sequencing was performed on *Salegentibacter mishustinae* using the Illumina MiSeq system. Paired-end reads averaged 250 bp in length, and the total read size of 3.84 Mb (clean data) was assembled using Velvet software version 2.8 (6). The assembled draft genome contained 3,775,942 bp in 38 large contigs with an average coverage of 190× and a G+C content of 36.5%. Maximum and average contig sizes were 639,574 bp and 99,367 bp, respectively.

A combination of Glimmer (7) and GeneMark (8, 9) was used to analyze the open reading frames (ORFs), and all predicted ORFs were then searched by BLAST against all proteins from complete microbial genomes using the NCBI Prokaryotic Genome Annotation Pipeline (10). The identification of tRNA genes and rRNA genes was performed by tRNAscan-SE version 1.21 (11) and RNAmmer version 1.2 software (12), respectively.

A total of 3,286 protein-coding genes were obtained, and 4 rRNAs and 41 tRNAs existed in the genome. Among the 3,286 putative protein-coding genes, 1,887 (57%) were assigned to 363 subsystem categories.

A detailed genomic inspection of strain KCTC 12263 revealed the presence of a denitrifying reductase gene cluster. In addition, a *Bacteroides* aerotolerance operon, a type I restriction-modification system, and a heavy metal efflux system were also found. The complete genes coding for flagella were not detected; however, two genes (*gldFG*) responding for gliding motility were observed.

Interestingly, one clustered regularly interspaced short palindromic repeat (CRISPR) was identified in the genome by using CRISPRFinder (13). CRISPR functions as adaptive immune systems against invaders such as viruses and plasmids (14–16). The CRISPR system in *Salegentibacter mishustinae* KCTC 12263 begins at position 587432 and ends at position 590615 in the whole genome (LKTP01000001), and has a conserved region (DR) of ATTCCAGACCATTCCAA to TTAGAAACTAGGATTGAAAC and 43 spacers. The CRISPR-Cas system in *Salegentibacter mishustinae* KCTC 12263 contains *cas8*, *cas7*, *cas5*, and *cas3* genes, which makes it a typical subtype I-B. The genes encoding information-processing subsystems Cas1, Cas2, and Cas4 are all present in the system and are thought to be involved in spacer integration during the adaption stage and thus active in repelling foreign genetic elements (17).

We reported the *Salegentibacter mishustinae* genome and provided primary information on its adaptation and metabolism. CRISPR-Cas system was identified in the genome and predicted to belong to subtype I-B and active, which shows the strategy against foreign nucleic acids of the strain. Further experimental evidences to support the prediction deduced from the genome sequence would provide a better understanding for the genus *Salegentibacter*.

### Nucleotide sequence accession numbers.

This whole-genome shotgun project has been deposited at DDBJ/EMBL/GenBank under the accession number LKTP00000000. The version described in this paper is the first version, LKTP01000000.
